# Postural Stability and Cognitive Performance of Subjects With Parkinson’s Disease During a Dual-Task in an Upright Stance

**DOI:** 10.3389/fpsyg.2020.01256

**Published:** 2020-07-29

**Authors:** Luis Morenilla, Gonzalo Márquez, José Andrés Sánchez, Olalla Bello, Virginia López-Alonso, Helena Fernández-Lago, Miguel Ángel Fernández-del-Olmo

**Affiliations:** ^1^Department of Physical Education, Faculty of Sport Sciences and Physical Education, University of A Coruña, A Coruña, Spain; ^2^Department of Biomedical Sciences, Medicine and Physical Therapy, Faculty of Physical Therapy, University of A Coruña, A Coruña, Spain; ^3^Department of Physical Education, Center of Higher Education Alberta Giménez, Palma, Spain; ^4^Department of Nursing and Physiotherapy, University of Lleida, Lleida, Spain; ^5^Department of Humanities, King Juan Carlos University, Móstoles, Spain

**Keywords:** dual-task, Parkinson’s disease, upright stance, postural sway, balance

## Abstract

**Background:**

The reviewed studies on center of pressure (COP) displacement in Parkinson’s disease (PD) subjects show important methodological differences and contradictory results with regard to healthy subjects. The dual-task paradigm method has been used to examine cognitive prioritization strategies to control concurrent postural and cognitive tasks. The motor requirements, such as pronouncing words, involved in the cognitive tasks used in double-task conditions could be related to the heterogeneity of the results.

**Research Objective:**

To compare postural sway and cognitive performance in subjects with PD and controls using a dual-task paradigm with a cognitive task free of motor demands. We tried to examine the prioritization strategy of PD patients regarding healthy adults to control for concurrent postural and cognitive tasks.

**Materials and Methods:**

25 subjects with PD and 20 healthy controls carried out a postural task under both single-task and dual-task conditions. The postural task was to stand as still as possible, with eyes first open and then closed. The dual-task condition added a concurrent cognitive task based on phoneme monitoring. COP displacement variables and cognitive performance were compared between the groups and within-subject factors were also examined.

**Results:**

PD participants showed higher COP displacement results than the controls. All participants shortened the mean sway radius in dual-task conditions compared with single-task conditions; only healthy subjects presented less transversal COP sway in dual-task conditions than in single-task conditions. The cognitive performance of PD patients on a phoneme monitoring task worsened when they carried it out while maintaining balance in a standing position compared to sitting. The opposite effect occurred in control subjects.

**Conclusion:**

This study confirms the negative influence of Parkinson’s disease on the control of standing stability, increasing the COP sway amplitude. The attentional demands of a postural task, such as standing balance, may be greater in PD patients than in healthy subjects. This would affect the performance of patients during dual-task conditions to be able to control a postural task while performing other cognitive tasks. In these conditions, cognitive performance would be negatively affected. These results suggest that subjects with PD, at least during initial disease stages, prioritize postural control over other concurrent tasks, as is also seen in healthy subjects.

## Introduction

Parkinson’s disease (PD) common motor symptoms, such as akinesia, rigidity, or resting tremors, are often early signs of the disease. However, postural stability differences between subjects with PD and healthy matches can be found even earlier than motor impairments become evident and sufferers need anti-parkinsonian medication (Mancini et al., 2012, 2011). The rise of motor symptoms is related with a worsening of balance control during basic activities like standing or walking, increasing the probability of unexpected falls (Ashburn et al., 2001).

Balance impairment can be quantified with a postural sway measure (Matinolli et al., 2007). The center of pressure (COP) displacement analysis is a procedure frequently used to examine the postural sway during unperturbed standing. The displacement of COP is not exactly equal to the displacement of center of gravity (COG), but it may reflect the net motor pattern that the central nervous system uses in the process of correction of the COG imbalance (Winter et al., 1990). Although clinicians usually don’t use posturography data to test standing stability in PD sufferers, several studies have shown the usefulness of some variables calculated from COP trajectory during unperturbed standing to characterize the stability changes caused by the disease (Rocchi et al., 2006; Blaszczyk et al., 2007; Mancini et al., 2012).

Despite these findings, studies comparing postural sway during an upright stance between PD sufferers and similar-age control subjects have reported contradictory results. Studies have shown both increased (Viitasalo et al., 2002; Schmit et al., 2006; Blaszczyk et al., 2007; Chastan et al., 2008; Blaszczyk and Orawiec, 2011; Mancini et al., 2012) and reduced (Horak et al., 1992; Ebersbach and Gunkel, 2011) postural sway in PD subjects, as well as no differences between the two groups (Schieppati and Nardone, 1991; Marchese et al., 2003; Holmes et al., 2010). It should be noted that among the reviewed studies there are significant differences in the methodology, such as the postural sway parameters recorded, the measurement instruments used, or the characteristics of the participants with PD or their matched controls.

Postural sway during a standing posture in PD subjects has been studied under a dual-task paradigm to explore the role of cognitive processes, with particularly attention paid to postural responses. Optimal performance in dual-task conditions needs cognitive strategies controlled by the executive system (Marois and Ivanoff, 2005). There is evidence of executive dysfunction in people with PD, even from early stages of the disease (Dirnberger and Jahanshahi, 2013). The review of Bloem et al. (2006) about PD and dual-tasks clearly shows how postural control of these subjects gets worse when a secondary concurrent task needs to be performed. Most of the reviewed studies focused on gait, while not many of them deal with balance during an upright stance. These researchers also indicate that prioritizing balance over other concurrent tasks is a common response in healthy young adults. Some of the studies reviewed by Bloem et al. (2006) would point out that this safe strategy is less frequent in older people. In addition, subjects with some degree of cognitive impairment, such as that which may be present in PD, Alzheimer’s, or Progressive Supranuclear Palsy, would not show a clear choice of balance control over any other concurrent cognitive task. An incorrect task prioritization could lead these subjects to hazardous behaviors during dual-task conditions that require control of posture or gait, increasing the risk of falling. Yogev-Seligmann et al. (2012) in a later review on dual-task control during gait develop a more complex approach about the prioritization strategy between motor and cognitive tasks that could be applied to any balance task. These authors provide a prioritization model based on two primary factors: one would be the postural reserve, based on sensory-motor health, and the other would be the cognitive estimation of the risk of the motor task. In PD patients, both postural reserve and risk estimation can be affected by the disease progression. Subjects with low postural reserve would prioritize balance control and safety over the performance of the cognitive task. In this sense, the level of difficulty in the balance task can influence the prioritization strategy. In healthy elderly people, it has been proven that increasing the difficulty of the standing balance can lead to the prioritization of the motor task over any cognitive concurrent task (Lion et al., 2014).

As in the case of single task studies, the results from dual-task studies are not conclusive. Some studies have found that the effects of a dual-task on postural sway is similar in sufferers and healthy subjects, either worsening stability (Morris et al., 2000; Barbosa et al., 2015; Fernandes et al., 2015) or not affecting it (Schmit et al., 2006). Others have reported inconsistent differences between PD and control subjects. In a study by Marchese et al. (2003), while counting backward aloud in multiples of three only PD subjects increased their center of pressure (COP) sway area, in contrast to the results reported by Holmes et al. (2010), where PD subjects showed smaller COP sway amplitudes than controls when recounting a monolog. Differences in experimental protocols and the cognitive dual-task could explain the heterogeneity of the results. More importantly, in all these previous studies the cognitive task involved a motor response in the form of the articulation of words. This may be a confounding factor since PD subjects have motor planning and programming difficulties (Stelmach et al., 1987; Weiss et al., 1997). In addition, it has been reported that changes in the various sway parameters that accompany the performance of secondary tasks may be related to the motor requirements of the task, such as those involved in articulating words (Dault et al., 2003). Therefore, and in order to attribute the changes in the COP during a quiet stance in PD subjects to attentional load, the cognitive task must avoid any motor components. However, to the best of our knowledge, there are no studies on PD that have used a cognitive dual-task meeting that requirement.

Furthermore, from a methodological point of view, any attempt to analyze the postural sway in people with PD should take into consideration the effect of two factors. One of them would be the use of antiparkinsonian medication and the other would be the aging process. Regarding the first one, the use of dopamine replacement medication (levodopa) reduces rigidity during ON state although, at the same time, a decrease in muscle tone during standing could worsen automatic postural responses to control balance (Horak et al., 1996). Moreover, motor fluctuations and dyskinesia are common side effects of the cumulative levodopa dosing as the disease advances (Hauser et al., 2006), leading to greater postural sway (Mancini et al., 2011). Different results have been obtained from the effect of this treatment on postural responses in subjects with the disease in an early and mild state. For instance, Beuter et al. (2008) found a decreased anteroposterior (A-P) and mediolateral (M-L) sway, and a reduced sway area in ON regarding OFF state. The study of Menant et al. (2011) also revealed a beneficial effect of levodopa on postural stability, with less A-P sway in ON state, although not to the level of healthy controls. The cognitive effects that dopaminergic medication may have on the executive function of PD patients should also be considered when observing dual-tasks with motor and cognitive components. Following the “dopamine overdose” hypothesis (Dirnberger and Jahanshahi, 2013), the positive effect of increased dopamine levels on motor and dorsolateral circuits of the striatum could at the same time overstimulate its ventral part, negatively affecting some cognitive functions mediated by orbitofrontal and limbic circuits. Concerning the second factor, the natural process of aging has been associated with a deterioration of stability in healthy adults (Woollacott, 2000). This process would be related with a greater body sway shown by older adults compared to young people during quiet standing (Lacour et al., 2008). Horak et al. (1992) suggested that elderly subjects tended toward parkinsonian-like increased stiffness with a decreased peak sway. Therefore, stability impairments in elderly PD sufferers caused by disease and its treatment could be confounded with the age-related worsening in postural stability.

Considering the above, the main goal of our study was to compare postural sway changes in both PD and control subjects using a dual-task paradigm with a concurrent non-motor task to facilitate a better understanding of the role of attentional demands on postural control in PD patients during ON state. Secondly, we aimed to confirm whether this neurodegenerative pathology, regardless of the patient’s age, could affect the control of balance in an upright position, increasing the attention demands of this basic motor task. Through the dual-task methodology, we tried to examine the hypothesis that subjects with PD do not give priority to postural control over performance in the concurrent cognitive task, showing some disability for the execution of multiple tasks. To fulfill this purpose, it was especially important to use a cognitive task whose performance could be analyzed under single and dual-task conditions.

## Materials and Methods

### Participants

Twenty-five subjects with PD (10 women, 15 men; mean age 57.6 ± 11.5) and twenty healthy controls (10 women, 10 men; mean age 59.1 ± 13.3) entered the study after giving their written informed consent according to the declaration of Helsinki. The patients were recruited from local Parkinson’s disease associations. Inclusion criteria for participants with PD was: diagnosis of idiopathic PD; absence of neurologic disorders other than PD; and absence of orthopedic, cardiovascular, auditory, or visual disturbances that could affect stability in upright stance. Healthy controls were included if they did not have a history of neurological pathology or other disease that could affect their standing stability. To admit a participant in the control group, it had to be possible to match them in terms of sex and age to a participant in the PD group. All study participants, both PD patients and control group subjects, were tested with the Mini-Mental State Examination (MMSE) (Folstein et al., 1975) to detect the possible presence of dementia and/or cognitive deficits. It was also found that were no significant differences between the average scores obtained in this test by each group. PD stage was rated by an experienced neurologist using the Hoehn and Yahr scale (H&Y) (Hoehn and Yahr, 1967) combined with the motor examination from the Unified Parkinson’s Disease Rating Scale (UPDRS-Section III) (Fahn and Elton, 1987). Both during the previous evaluation and during the postural and cognitive tasks, each patient was measured in ON state, at the approximate peak of medication effect (45 min-1 h after medication intake). The demographic, anthropometric, and cognitive characteristics of the participants are summarized in Table 1. Table 2 shows the main clinical characteristics of the PD participant group. The Ethics Committee of our institution approved the experimental protocol (ref. 346).

**TABLE 1:** Participant characteristics data.

	*N*	Age (years)	Weight (kg)	Height (cm)	MMSE
PD patients	25	57.6 ± 11.5	78.7 ± 14.9	166.8 ± 7.7	29.6 ± 0.8
	10♀/15♂				
Controls	20	59.1 ± 13.3	72 ± 9.7	163.8 ± 7.9	29.4 ± 0.9
	10♀/10♂				
*p-*value between groups		0.695	0.078	0.210	0.435

*Values are Mean ± Standard Deviation, t-test analysis for group differences. N, sample; MMSE, Mini-Mental State Examination; H&Y, Hoehn and Yahr scale; UPDRS-III, Unified Parkinson’s Disease Rating Scale, section III.*

**TABLE 2:** Clinical characteristics of the PD patients’ group.

Patient number	Sex	Age	Disease duration (years)	H&Y (1 – 5)	UPDRS-III (0 – 56)	Medication per day (mg)	LED (mg)
1	F	40	1	1	4	Levodopa/Carbidopa 300/75, Rasagiline 1, Rotigotine 6	580
2	M	66	1,5	1	7	Levodopa/Carbidopa 375/37.5, Rasagiline 1	475
3	M	37	2	1	9	Rasagiline 1	100
4	M	70	2	1	8	Levodopa/Carbidopa 375/37.5, Rasagiline 1	475
5	M	56	2	1,5	12	Pramipexole 2.1	210
6	F	46	1	2	23	Levodopa/Carbidopa 150/37.5, Rasagiline 1, Pramipexole 3.15	565
7	F	39	2,3	2	18	Levodopa/Carbidopa 375/93.75, Entacapone 600, Rasagiline 1	673
8	M	45	3	2	11	Levodopa/Carbidopa 225/56.25, Entacapone 600, Rasagiline 1, Rotigotine 4	643
9	M	67	3	2	15	Levodopa/Benserazide 500/125, Rasagiline 1, Pramipexole 2.64	864
10	M	62	4	2	12	Levodopa/Carbidopa 150/37.5, Entacapone 600, Pramipexole 3.15	663
11	M	50	5	2	19	Levodopa/Carbidopa 300/75, Rasagiline 1, Rotigotine 8	640
12	F	51	5	2	36	Levodopa/Benserazide 600/150	600
13	M	58	6	2	23	Levodopa/Carbidopa 500/125, Rasagiline 1, Rotigotine 4	720
14	M	60	6	2	12	Levodopa/Carbidopa 800/200, Entacapone 800, Pramipexole 3.15	1379
15	M	55	6	2	23	Levodopa/Carbidopa 225/56.25, Entacapone 600, Rasagiline 1, Ropinirole 20	923
16	F	61	6	2	23	Levodopa/Carbidopa 500/50, Pramipexole 3.15	815
17	M	60	7	2	13	Levodopa/Carbidopa 500/125, Ropinirole 12, Trihexyphenidyl 2	740
18	M	63	8	2	21	Levodopa/Carbidopa 750/187.5, Entacapone 1000, Rasagiline 1, Pramipexole 3.15	1580
19	M	68	1	2,5	16	Levodopa/Carbidopa 150/37.5, Entacapone 600, Rasagiline 1	448
20	M	80	6	2,5	29	Levodopa/Benserazide 500/125	500
21	F	46	6	2,5	23	Levodopa/Carbidopa 200/50, Levodopa/Benserazide 550/137.5, Rotigotine 6, Rasagiline 1, Amantadine 200	1230
22	F	54	6	2,5	25	Levodopa/Carbidopa 400/100, Entacapone 800, Rotigotine 12	1024
23	F	66	12	2,5	12	Levodopa/Carbidopa 200/50, Levodopa/Benserazide 550/137.5, Rotigotine 6, Rasagiline 1, Amantadine 200	1230
24	F	62	2	3	31	Levodopa/Carbidopa 600/150, Entacapone 600, Rotigotine 6, Pramipexole 3.15	1293
25	F	79	7	3	35	Levodopa/Carbidopa 400/50, Pramipexole 0.18	418
Mean		57,6	4,42	2	18,4		751,52
SD		11,5	2,71	0,56	8,69		358,63

*F, female; M, male; H&Y, Hoehn and Yahr scale; UPDRS-III, Unified Parkinson’s Disease Rating Scale, section III; LED, Levodopa Equivalent Dose (Tomlinson et al., 2010).*

### Postural and Cognitive Tasks

For the standing task, and to record balance data, each subject stood on a tri-axis force plate (Kistler 9286BA), with their feet hip-width apart, at an angle of 30 degrees, with their arms hanging next to their body. The point location of the vertical ground reaction force vector, the COP, and its displacement were calculated using a BTS SMART Analyzer© and BTS Sway© software (BTS Bioengineering) with a sampling rate of 100 Hz.

The cognitive task was designed following a phoneme monitoring paradigm (Connine and Titone, 1996). This kind of task has been previously used to demand attention in several studies of PD and gait (Yogev et al., 2005; Springer et al., 2006; Hausdorff et al., 2008; Bello et al., 2013). For this task, subjects were required to listen to a story, using earphones, and count how many times two particular pre-established words appeared. The reasons for choosing a phoneme monitoring task as the cognitive task were, first of all, to avoid the “contaminant” effect of speech or other response motor mechanisms and secondly, to establish a cognitive task that demanded attention evenly during the dual-task. The phoneme monitoring task is not a perceptual task and therefore cannot provide useable information for postural control. We also considered the proved postural effect of auditory cognitive tasks (Deviterne et al., 2005; Riley et al., 2005).

### Procedure

Each participant attended two individual sessions. In the first session, the postural and cognitive tasks were explained to the participants and they tried them in the same conditions in which they would be performed in the second session. The second session, 1 day later, was the evaluation session.

Each session involved five trials. The first trial was to perform the cognitive task while seated in a chair. This data was used as the baseline for cognitive performance. The next four trials were assigned to test cognitive-postural tasks in four different conditions: (a) single task, only standing, with open eyes (STOE); (b) single task with closed eyes (STCE); (c) dual-task, standing plus cognitive task, with open eyes (DTOE); and (d) dual-task with closed eyes (DTCE). In open-eye conditions, the subjects had to look at an eye-level black target on a white screen placed 1 m in front of them. A 60 s period was recorded for each trial under each condition during the second session. To control for any possible effects due to the sequence of tests, each participant performed the four tasks in a randomized order. Additionally, the different texts used as stories in the cognitive tasks were counterbalanced among sessions and participants. No text was repeated in the different essays performed by each participant in the two sessions.

Before each trial, the same instructions were given to all participants. For single-task conditions, the participants were asked to stand on the platform as still as possible. For dual-task conditions, they were encouraged to do both cognitive and standing tasks as well as possible. The instructions given to complete the dual-task asked the participants to try to identify and mentally count how many times each word a specific word had been mentioned.

### Data Analysis

The following variables were calculated from COP displacement: (1) trace length, as the total length of path traced by COP on the force plate (mm). This variable allowed us to know the mean speed (mm⋅s^–1^) as the average speed of COP during the 60 s of each recorded trial; (2) area (mm^2^), as the total area covered by the COP displacement; (3) radius, as the mean distance (mm) from COP to center of gravity during COP sway; (4) M-L and A-P sway, as the mean COP displacement (mm) along the *x* and *y* axes, respectively, with respect to the center of the force plate; and (5) M-L and A-P ranges, as the difference, respectively, between the maximum and minimum values (mm) of COP sway along the *x* and *y* axes.

For the cognitive task, the error in phoneme monitoring was obtained. The number of errors in each cognitive task was established as the difference between the number of words counted by the subject and the correct number. The ratio of errors to total target phonemes appearing in the story was used to analyze cognitive task performance.

### Statistical Analysis

To rule out any possible initial differences between the groups, a *t*-test was performed on age, the anthropometric variables (height and weight), and the cognitive evaluation (Mini-Mental State Examination) of the participants.

For the COP displacement variables, a mixed design of repeated measures analysis of variance (ANOVA) was performed to examine the potential differences between the two groups (PD participants and healthy controls) with respect to the effect of within-subject factors: vision (open or closed eyes) and task (single or dual-task), as well as possible interaction effects (three-way ANOVA). Data distribution normality was checked beforehand, as was the sphericity and homogeneity of variance. A measure of effect size for the statistically significant effects was obtained using partial Eta squared values (η_*p*_^2^). When the *post hoc* analysis showed a significant difference in the pairwise comparisons, effect sizes were calculated using Cohen’s *d* for the within-subject differences (e.g., single vs. dual-task for healthy controls group).

In the introduction it was mentioned how aging is a factor that affects postural control. It has also been verified how it can affect dual-task management (Woollacott and Shumway-Cook, 2002; Lacour et al., 2008; Doumas et al., 2009; Olivier et al., 2010). Consequently, in the study it was important to be able to clearly separate the effect of Parkinson’s disease on the stability of the aging effect. Although there were no significant differences in the mean age of each group (Table 1), the age ranges were not the same (51 vs. 43), with greater variability in the control group. So, we decided to include age as a covariate to control for its influence on the results of the study participants. The use of age as a covariate was intended to reduce within-group error variance, assuming that some variability in the stability data of each group could be given by the age variable. None of the contrasts were made with age as a covariate in their interaction with the within-subject factors: vision (open or closed eyes) and task (single or dual-task) were significant. However, when removing this covariate from the analysis, the ANOVA did not produce the same results, showing significant effects of the vision and task factors on most of the variables of the COP displacement. In our statistical treatment, we opted for a more conservative approach that would allow for a more accurate determination of the effects of inter and intra-subject factors. Therefore, it was decided to keep age as a covariate in the statistical analysis.

To analyze the cognitive performance of the participants under dual-task conditions, a further repeated measures ANOVA was planned (two-way). Group (participant with PD or healthy control) was once again used as a between-groups factor. For the cognitive task, the three conditions of within-subject factor were sitting on a chair (baseline), during DTOE, and DTCE conditions. The variable that expressed cognitive performance, the ratio errors/phonemes, failed the data distribution normality and variance homogeneity assumptions. A non-parametric ANOVA-type test was conducted. This statistic allows the same analysis as a traditional ANOVA (i.e., the effect of each factor and the interaction between them) but is based on the use of ranks for calculating the so-called relative marginal effects (Noguchi et al., 2012). When a significant interaction was detected, paired comparison between groups was applied by using the Mann–Whitney *U*-test and paired comparison within the groups was determined with the Wilcoxon Signed-Rank test with Bonferroni’s adjustment. A measure of effect size was included by calculating Cohen’s *d* from the *U* and *Z* values reported by the non-parametric test used in pairwise comparisons (Fritz et al., 2012; Lenhard and Lenhard, 2016).

All statistical analyses were performed using SPSS software (IBM SPSS Statistics, release 20.0). A *p*-value of ≤0.05 was considered statistically significant.

## Results

The *t*-test showed no significant differences between groups for age, weight, height, and cognitive evaluation (Table 1). The means and standard deviations, and ANOVA results for COP displacement variables, are shown in Tables 3, 4, respectively.

**TABLE 3:** COP displacement variable data recorded in the four test conditions for PD patients and control groups.

	Control group				PD patient group			
	ST		DT		ST		DT	
	OE	CE	OE	CE	OE	CE	OE	CE
Trace length (mm)	1433.42 ± 311.66	1535.72 ± 270.34	1460.89 ± 309.23	1570.06 ± 334.99	1484.12 ± 316.47	1704.42 ± 407.54	1519.86 ± 424.89	1727.27 ± 577.12
Area (mm^2^)	1958.87 ± 626.61	2291.25 ± 1110.07	1821.46 ± 825.60	2007.67 ± 1136.01	3030.70 ± 1254.25	4011.22 ± 1637.79	2683.34 ± 979.07	3473.88 ± 1371.51
Radius (mm)	4.81 ± 1.67	5.24 ± 1.98	4.34 ± 4.31	4.31 ± 1.45	6.75 ± 2.35	7.69 ± 1.95	5.78 ± 1.38	6.43 ± 1.33
A-P sway (mm)	40.49 ± 26.89	46.08 ± 24.61	44.88 ± 28.65	40.86 ± 30.90	69.28 ± 31.48	67.49 ± 31.29	64.06 ± 35.65	63.26 ± 24.22
M-L sway (mm)	6.85 ± 4.91	5.74 ± 4.67	4.49 ± 4.69	4.60 ± 3.90	6.05 ± 4.47	6.60 ± 5.31	7.35 ± 3.85	6.47 ± 4.33
A-P range (mm)	22.81 ± 7.55	28.78 ± 9.90	21.67 ± 8.58	23.43 ± 7.94	31.32 ± 13.25	36.98 ± 9.46	27.43 ± 8.38	31.77 ± 8.69
M-L range (mm)	15.71 ± 5.42	17.41 ± 8.51	14.20 ± 5.76	15.62 ± 7.33	23.74 ± 7.25	29.81 ± 9.12	21.44 ± 5.26	28.05 ± 9.12

*Values are Mean ± Standard Deviation. PD, Parkinson’s disease; ST, single task; DT, dual-task; OE, open eyes; CE, closed eyes; A-P, anteroposterior; M-L, mediolateral.*

**TABLE 4:** ANOVA results for COP displacement variables.

	Group	Vision	Group × Vision	Task	Group × Task	Vision × Task	Group × Vision × Task
Trace length (mm)	ns	ns	ns	ns	ns	ns	ns
Area (mm^2^)	F_1_,_42_ = 19.203 *p* < 0.001 η_*p*_^2^ = 0.314	ns	F_1_,_42_ = 6.637 *p* = 0.014 η_*p*_^2^ = 0.136	ns	ns	ns	ns
Radius (mm)	F_1_,_42_ = 25.822 *p* < 0.001 η_*p*_^2^ = 0.381	ns	ns	F_1_,_42_ = 6.128 *p* = 0.017 η_*p*_^2^ = 0.127	ns	ns	ns
A-P sway (mm)	F_1_,_42_ = 8.304 *p* = 0.006 η_*p*_^2^ = 0.165	ns	ns	ns	ns	ns	ns
M-L sway (mm)	ns	ns	ns	ns	F_1_,_42_ = 4.454 *p* = 0.041 η_*p*_^2^ = 0.096	ns	ns
A-P range (mm)	F_1_,_42_ = 13.832 *p* = 0.001 η_*p*_^2^ = 0.248	ns	ns	ns	ns	ns	ns
M-L range (mm)	F_1_,_42_ = 29.068 *p* < 0.001 η_*p*_^2^ = 0.409	ns	F_1_,_42_ = 9.938 *p* = 0.003 η_*p*_^2^ = 0.191	ns	ns	ns	ns

*A-P, anteroposterior; M-L, mediolateral; ns, not significant; η_p_^2^, partial Eta squared.*

### Effects on Upright Standing Task

The data analysis revealed significant effects on several COP displacement parameters (Table 4). The three-way ANOVA showed a main effect of group for area (*p* < 0.001, η_*p*_^2^ = 0.314), radius (*p* < 0.001, η_*p*_^2^ = 0.381), A-P sway (*p* = 0.006, η_*p*_^2^ = 0.165), A-P range (*p* = 0.001, η_*p*_^2^ = 0.248), and M-L range (*p* < 0.001, η_*p*_^2^ = 0.409), with higher sway results being recorded for participants with PD than control participants.

**FIGURE 1:**
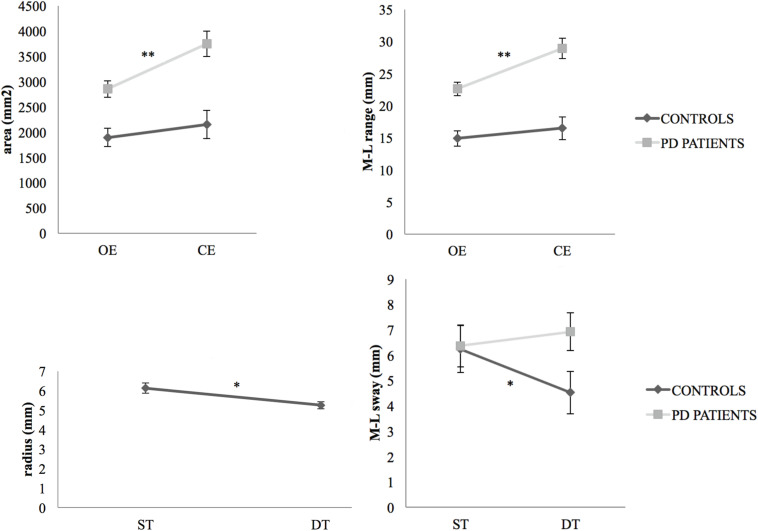
Vision and task effects on COP displacement variables. Changes appeared in the total area covered by the COP displacement and in the M-L range of COP sway (mean values with standard error bars) during a dual-task performed from the open eyes condition to closed eyes, for PD patients (**p < 0.0001) and controls. Changes (mean values with standard error bars) were recorded in the radius of COP sway for all participants (*p < 0.05) as well as in the M-L sway of COP for controls (*p < 0.05) and PD patients, from single-task (standing task only) to dual-task (standing plus cognitive task) conditions. PD, Parkinson’s disease; OE, open eyes; CE, closed eyes; ST, single task; DT, dual-task.

The analysis revealed a group × vision interaction effect for area (*p* = 0.014, η_*p*_^2^ = 0.136) and M-L range (*p* = 0.003, η_*p*_^2^ = 0.191). Specifically, *post hoc* analyses showed, only for PD subjects, a significant increase in area (*p* < 0.001, Cohen’s *d* = 0.662) and M-L range (*p* < 0.001, Cohen’s *d* = 0.808) from tasks performed with open eyes to those performed with closed eyes (Figure 1). For radius, the ANOVA detected a cognitive task effect. In the standing task, all participants, both controls and PD patients, shortened their mean sway radius under dual-task conditions compared with single-task conditions (*p* = 0.017, η_*p*_^2^ = 0.127). For M-L sway, the ANOVA showed a group × task interaction effect (*p* = 0.041, η_*p*_^2^ = 0.096). *Post hoc* analysis indicated that the healthy controls showed less mean transversal COP displacement during the dual-task than during the standing only task (*p* = 0.038, Cohen’s *d* = 0.387, Figure 1).

### Effects on Cognitive Task

**FIGURE 2:**
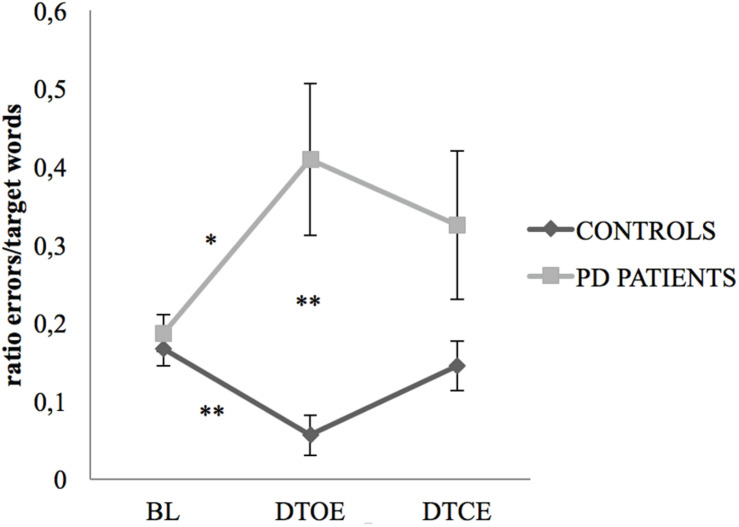
Task effect on cognitive performance. Changes appeared in cognitive task performance (mean values with standard error bars) from single-task conditions (cognitive task conducted by seated participants) to the dual-task with open eyes (for PD patients, *p = 0.039, and controls, **p = 0.009) and the dual-task with closed eyes. Differences were recorded between the groups in cognitive task performance in the dual-task with open eyes condition (**p < 0.01). PD, Parkinson’s disease; BL, cognitive baseline; DTOE, dual-task open eyes; DTCE, dual-task closed eyes.

The non-parametric ANOVA-type analysis showed a main effect of group for cognitive performance (*F*_1_,_42_._99_ = 10.13, *p* = 0.001), as well as a significant group × task interaction (*F*_1_._88_,_∞_ = 7.41, *p* < 0.001). The Mann–Whitney *U*-test detected significant differences between the groups only for the dual-task with open eyes (*U* = 95.00, *p* < 0.001, Cohen’s *d* = 1.243). Under this condition, PD subjects showed a higher error ratio than control participants (Figure 2). For within-group pairs comparisons, adding the phoneme monitoring task to the standing task had a different principal effect on sufferers than on healthy controls. While the ratio of errors/phonemes in PD subjects rose from the baseline to the dual-task with open eyes (Wilcoxon Signed Rank test, *Z* = −2.489, *p* = 0.039 after Bonferroni’s adjustment, Cohen’s *d* = 0.752), control subjects showed the opposite behavior, reducing this ratio (Wilcoxon Signed Rank test, *Z* = −2.938, *p* = 0.003 after Bonferroni’s adjustment, Cohen’s *d* = 1.049).

## Discussion

The study results clearly show the existence of differences between people with PD and healthy similarly aged controls when it comes to upright stance stability. The posturography data records showed these differences in COP behavior as a greater sway amplitude. In addition, the participants with PD presented a poorer performance in the phoneme monitoring task in the standing position, suggesting a prioritization of postural control over the other concurrent task.

### Upright Stance Stability

The greater sway area and sway displacement in the A-P and M-L directions of PD participants compared with the control subjects are in line with previous studies (Schieppati et al., 1994; Viitasalo et al., 2002; Raymakers et al., 2005; Blaszczyk and Orawiec, 2011). These results would reinforce the theory that PD increases the amplitude of COP sway during a standing position. M-L sway has been related with a tendency to fall in PD subjects (Blaszczyk et al., 2007) and has been proposed as a good indicator of disease progression (Mancini et al., 2012). It has been suggested that the larger M-L COP displacement in PD subjects could be a compensatory strategy related to the PD subjects’ restriction of movement in the A-P direction (Mitchell et al., 1995). However, studies of A-P sway in PD subjects have reported inconsistent results, showing both lower (Horak et al., 1992; Ebersbach and Gunkel, 2011) and higher (Blaszczyk et al., 2007) results, in addition to no differences in A-P sway between PD and control subjects (Mitchell et al., 1995). This discrepancy could result from the different features of the PD subjects that participated in each study, since COP displacement in the A-P direction can be affected by axial rigidity (Horak et al., 2005) and thus the disability level caused by the disease.

Privation of vision led to an increase in COP area and M-L range in PD subjects while it appeared to have no significant effects on the control subjects in any of the COP measurements. This finding is in line with previous studies, showing that the exclusion of vision causes greater stability deterioration in PD patients than controls (Schieppati et al., 1994; Marchese et al., 2003; Blaszczyk et al., 2007; Blaszczyk and Orawiec, 2011). This is consistent with the poor use of proprioceptive feedback due to an increase in proprioceptive loop noise with abnormal feedback gain (Maurer et al., 2004; Konczak et al., 2009).

### Dual-Task Performance

During the dual-task, all the participants reduced their COP radius, behavior already observed in young and elderly subjects (Andersson et al., 2002; Swan et al., 2004; Deviterne et al., 2005; Riley et al., 2005; Huxhold et al., 2006; Siu and Woollacott, 2007). In addition, the control subjects significantly reduced their COP sway in the M-L direction, as reported in previous studies (Andersson et al., 2002; Deviterne et al., 2005; Riley et al., 2005). However, M-L sway remained unaffected in the PD subjects. This result supports the premise of Mitchell et al. (1995) that PD sufferers tend to increase M-L sway as a strategy for reducing sway amplitude in the most threatening direction, the A-P plane. We can speculate that, while the reduced M-L sway in control subjects for the dual-task condition may reflect more automatic posture control, in PD subjects the unaffected M-L sway could indicate a restriction when it comes to freeing the posture from a more conscious control. In other words, if PD reduces the patients’ confidence in standing balance control, they could increase postural stiffness, resulting in less COP sway radius than the controls, to release attentional resources for cognitive performance during a dual-task. But the impairment in the basal ganglia circuitry caused by PD could hamper the automatic control of standing balance, which would lead patients to need more conscious postural control and, therefore, to be more demanding of attention. This strategy would give more importance to open-loop control mechanisms with long-latency postural responses, preventing the reduction of COP sway in the M-L plane during dual-task conditions.

The previous assumption would be supported by the results of the cognitive task of our study. Woollacott and Shumway-Cook (2002) pointed out that the changes in the cognitive task during the dual-task regarding the single-task condition could contribute information about the attentional demands of the postural task. We observed different behavior between PD patients and controls in cognitive performances during the dual-task. The score in the phoneme monitoring task was significantly worse in PD subjects for the dual-task condition (standing + cognitive task) compared with the cognitive task baseline condition. It is therefore likely that PD subjects allocate more attention to maintaining the standing position, to the detriment of the cognitive task. These results would not support the hypothesis of the introduction, according to which the subjects with PD would not prioritize stance balance over other concurrent tasks, choosing a “posture second” strategy. Our PD participants would have opted for a “posture first” strategy, ensuring stability control rather than being successful on the cognitive task, showing behavior similar to that of healthy adults (Bloem et al., 2006) or the elderly (Lion et al., 2014). However, it is possible that, with more advanced PD subjects than those in our study (e.g., 4 or 5 H&Y stages), the “posture second” strategy may prevail. In addition, the nature of the cognitive task used in this work could explain this discrepancy. The use of phoneme monitoring tasks avoids any confounding factors in PD subjects associated with cognitive tasks involving word articulation, and may therefore provide a better understanding of the attentional demands of postural tasks in PD subjects.

An unexpected result was the fact that the control subjects improved their cognitive task score during the standing balance task, when most studies show a deterioration in cognitive results for the dual-task, even when the postural task was as unchallenging as an unperturbed standing task (Lajoie et al., 1993; Mitra, 2003; Siu and Woollacott, 2007). A possible explanation could be the role of arousal as a mediating factor between postural and cognitive tasks (Andersson et al., 2002). According to Yerkes–Dodson law, the difficulty of the cognitive task could modulate the effect of arousal over the standing task (Huxhold et al., 2006), improving the former when the cognitive load of the dual-task is low but deteriorating it when the load increases. In other words, for the control subjects the standing task would enable good automatic control with low attentional demands, raising the arousal level enough to improve the cognitive performance during the dual-task. In contrast, in the PD patients’ group, the higher attention load of the standing task with less automatic control during the dual-task would result in a worsening of the cognitive results. In Yogev et al. (2005), subjects with PD performed as well as their healthy controls on a phoneme monitoring task while walking at a comfortable pace. Perhaps for PD patients, trying to remain still during a standing task may involve greater attention requirements than walking at an easy pace. To the best of our knowledge, this is the first study to evaluate the cognitive performance of PD subjects during a standing task.

Among the limitations of the study, we want to highlight the sample size and the level of homogeneity in the clinical characteristics of the PD patients’ group. Having a greater number of participants with PD would allow the sample to be stratified into several groups of patients according the disease stage (H&Y), the use of medication (LED), or the degree of motor impairment (UPDRS-III). In relation to this last factor, the characterization of PD participants in the two most widely used clinical phenotypes, dominant tremor versus postural instability/gait difficulty (Stebbins et al., 2013), would allow for a better understanding of the influence of PD on attentional demands of control of standing stability. In this sense and considering future studies, complementing the measures of the COP in the time domain with the measures in the frequency domain will allow us to use methods such as the fast Fourier transformation and the wavelet waveform for a more objective differentiation of the two motor subtypes (Rezvanian et al., 2018).

## Conclusion

This study confirms the negative influence of Parkinson’s disease on the control of standing stability, increasing the COP sway amplitude. The attentional demands of a postural task such as standing balance may be greater in PD patients than in healthy subjects. This would affect the performance of patients during dual-task conditions when they have to simultaneously control this postural task together with other cognitive tasks. In these conditions cognitive performance would be negatively affected. These subjects, at least during initial disease stages, possibly prioritize postural control over any concurrent task.

## Data Availability Statement

The datasets generated for this study are available on request to the corresponding author.

## Ethics Statement

The studies involving human participants were reviewed and approved by the University of A Coruña Research and Teaching Ethics Committee. The patients/participants provided their written informed consent to participate in this study.

## Author Contributions

LM and MF: conceptualization. LM, GM, JS, OB, VL-A, and MF: methodology. LM: formal analysis, writing – original draft, and visualization. LM, GM, and JS: investigation. LM, GM, JS, OB, VL-A, HF-L, and MF: writing – review and editing. MF: supervision, project administration, and funding acquisition. All authors contributed to the article and approved the submitted version.

## Conflict of Interest

The authors declare that the research was conducted in the absence of any commercial or financial relationships that could be construed as a potential conflict of interest.
